# Chronic exertional compartment syndrome of the forearm in a professional drummer: Case Report on the interest of botulinum toxin injection after surgery

**DOI:** 10.3389/fspor.2026.1680887

**Published:** 2026-02-05

**Authors:** Margaux Temperelli, Edem Allado, Oriane Hily, Julia Guirlinger, Benoit Pierret, Jean Paysant, Bruno Chenuel, Lionel Athlani, François Dap, Mathias Poussel

**Affiliations:** 1 CHRU-Nancy, University Centre of Sports Medicine and Adapted Physical Activity, Nancy, France; 2 DevAH, Department of Physiology, University of Lorraine, Nancy, France; 3 Institut Régional de Médecine Physique et de Réadaptation de Nancy, UGECAM du Nord-Est, Nancy, France; 4 CHRU-Nancy, Department of Hand Surgery, Plastic and Reconstructive Surgery, Centre Chirurgical Émile Gallé, Nancy, France

**Keywords:** botulinum toxin injection, case report, chronic exertional compartment syndrome, forearm, professional musician, surgery

## Abstract

Chronic exertional compartment syndrome (CECS) is a rare condition usually affecting the lower leg, but can also involve the upper arm. This exercise-induced pathology is considered an overuse injury characterized by increased pressure in a muscle compartment. Forearm CECS is mostly observed in sports requiring prolonged, repetitive and energetic gripping, but also in musicians. The main symptoms are ischemic pain, stiffness, reduced muscle strength and paresthesia. We report here the unique case of forearm CECS in a professional drummer, successfully treated with injection of botulinum toxin following a post-surgical recurrence. The multiple intramuscular pressure measurements of the forearm performed during a 2 years follow-up suggest the efficacy of toxin botulinum injections on stiffness, pain and functional limitation in a patient with upper limb CECS following post-surgical recurrence. Injection of botulinum toxin should therefore be considered in CECS.

## Introduction

Chronic exertional compartment syndrome (CECS) is a rare condition first described in the forearm in 1983 (1). CECS usually affects the lower leg, but can also involve shoulder, arm, forearm, hand, thigh and foot (2). This exercise-induced pathology is an overuse injury characterized by increased pressure in a muscle compartment. The forearm is made up of 4 muscular compartments surrounded by fascia with poorly extensible mechanical properties: the superficial and deep anterior compartments, the lateral compartment and the posterior compartment. Symptoms include ischemic pain, stiffness, reduced muscle strength and paresthesia (forearm, hand, fingers). The condition resolves spontaneously without sequelae on cessation of exercise. Forearm CECS is observed in individuals practicing an activity requiring prolonged, repetitive and energetic gripping: cyclists, gymnasts (3), rowers (4), motocross (5) or motorcycle riders (6), weightlifters (7), but also musicians: pianist, bassist, violinist (8, 9). The diagnosis, primarily based on clinical findings, is confirmed by dynamic measurement of intra-compartmental pressure: resting pressure ≥15 mmHg and/or pressure ≥30 mmHg 1-minute post-exercise and/or pressure ≥20 mmHg 5-minute post-exercise (10). However, it must be appreciated that these criteria were originally based on values in suspected lower leg CECS. Others authors propose pressures between 15 and 30 mm Hg during recovery after maximal stress testing are also highly suggestive of CECS, as well as a pressure above 30 mmHg (11, 12). We report here the unique case of forearm CECS in a professional drummer, successfully treated with injection of botulinum toxin following a post-surgical recurrence.

## Case report

**Figure 1:**
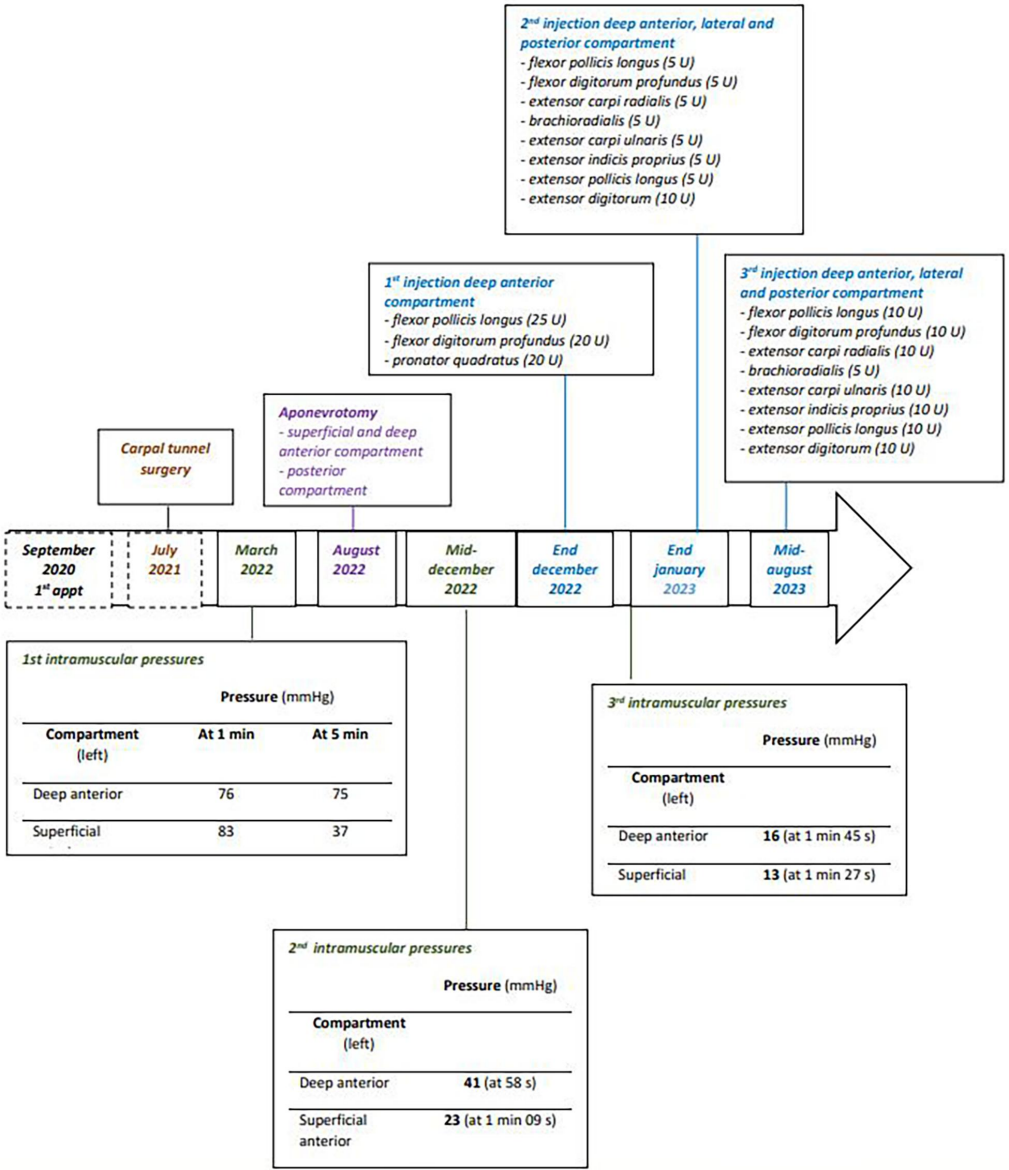
Timeline showing the results of the post exertion intramuscular pressures assessments and the doses of botulinum toxin injected in the symptomatic muscles.

In January 2022, a 51-year-old professional drummer presented in our clinic with a recurrent pain when playing, mainly affecting the left upper limb. Symptomatology had begun in 2014, with the intensification of his practice (15 h a week). Initially, pain affected the thumb and index finger, but then spread to all the fingers and the extensors of the forearm. Differential diagnoses were eliminated, in particular the absence of cervicothoracic outlet syndrome (no blockage or compression at the dynamic arterial doppler ultrasound test) or cervical disc herniation (normal cervical MRI). The patient described constrict pain in the left forearm, initially only occurring during intensive drumming practice, but then also present during daily activities (such as writing or brushing teeth). The intensity of the pain led the patient to stop current activity, allowing a regression of pain within 10–15 min. The physical examination at rest was normal. In March 2022, intramuscular pressure measurements of the left forearm were performed (after exercises that reproduce the patient's pain) confirming the diagnosis of CECS (Figure 1) according to Pedowitz's criteria. Considering the clinical presentation, the patient's profession and the confirmation of CECS for the superficial and deep anterior compartment as well as for the posterior compartment, a surgical management (aponeurotomy of the 3 compartments) was carried out at the end of August 2022 (in accordance of the patient's choice). There were no post operative complications. The scars were soft and non-adherent. The patient continued with additional self-administered stretching and flexibility exercises for the upper limbs. Fifteen days after surgery, the patient was able to use his forearm with a sensation of liberation. At 10 weeks post-surgery, discomfort had decreased without disappearing completely. When using the drums, the patient reported a feeling of stiffness of the extensor apparatus, and some pain or even cramps in the fingers of the left hand. Due to the suspected recurrence and in order to assess the effects of the surgical procedure, new pressure measurements were achieved mid-December 2022. Measurements showed an overall reduction in pressure, but still positive values (>30 mmHg) for the deep anterior compartment were observed (Figure 1). Given the recurrence of CECS, a non-invasive treatment was proposed after obtaining patient's consent. Patient then (end of December 2022) underwent injections of botulinum toxin (Xeomin®) under electrostimulation guidance in the muscles concerned: namely, 25 units in the flexor pollicis longus, 20 units in the flexor digitorum profundus and in the pronator quadratus. Stretching through self-exercises was encouraged. One month following botulinum toxin injection, the patient reported a regression of pain in the anterior compartment of the forearm but also a temporary weakness of his fingers (rated 3/5 on the MRC scale) in the week after the injections. No other side effects were reported, including no post-injection muscle spasms. Even if improved by the injections, he had to use more the posterior and lateral muscles to compensate for weak muscles and this trigger more pain in these compartments. As a result, the patient was able to resume drum lessons but in a fragmented way and did not add his recreational drumming practice. Daily activities were not impacted. In March 2023, pain reappeared mainly in the deep anterior, lateral and posterior forearm compartments, associated with stiffness and cramps in the posterior compartment and significant contractures in the extensor indicis proprius, leading to further injections of botulinum toxin (Xeomin®). Before the second series of injections, we decided to carry out new pressure measurements in order to adjust the doses of botulinum toxine. The third series of pressure measurements showed a clear reduction of the injected deep anterior compartment (Figure 1; 16 mmHg vs. 41 mmHg). Given the relatively low pressure (<30 mmHg) (Figure 1) and the risk of muscle weakness, the doses administered were lowered: 5 units in the flexor pollicis longus, flexor digitorum profundus, brachioradialis, extensor carpi radialis, extensor carpi ulnaris, extensor indicis proprius, extensor pollicis longus, and 10 units in the extensor digitorum. Three weeks post-injection, the patient described an almost complete improvement in his symptomatology, with the possibility of resuming his professional and leisure musical activities. He described a complete absence of muscle weakness with these lower doses. However, 6 weeks post-injection (May 2023), the patient described a gradual return of his muscular pain during activities, with a symptomatology almost identical to the pre-injection in July 2023. Mid-August 2023, given to the transient efficacy of the previous injections, new botulinum toxin injections were performed in the deep anterior, lateral and posterior compartments of the left forearm with higher doses: 10 units in the flexor pollicis longus, the flexor digitorum profundus, the extensor carpi radialis, the extensor carpi ulnaris, the extensor indicis proprius, the extensor pollicis longus, the extensor digitorum, and 5 units in the brachioradialis muscle. Slightly higher doses were tried, given the absence of muscle deficit, in the hope of more lasting effectiveness from the toxin injections.

At the end of November 2023, the patient described a regression of pain and reduced stiffness, with the possibility of resuming leisure and professional musical activities with no limitation. At the beginning of January 2024, the patient still described an improvement post-botulinum toxin injection, with no functional limitation in his activities. We advise him to continue stretching and self-exercises over the long term, and remain at his disposal in the event of an increase in pain or functional limitation.

## Discussion

The treatment of forearm CECS can be either conservative or surgical. Conservative treatment is based on activity modification, pain control and rehabilitation (13), with significant reduction in pain up to 80% of patients (5). Patients (14) or top-level athletes (8) often reject the option of reducing or even stopping practice. When symptoms persist after 3 months of conservative treatment, fasciotomy surgery is indicated (5). Three main techniques are available: open, mini-open and endoscopic, with open fasciotomy long considered the gold standard (13). However, as the invasive nature of this procedure prevents a quick return to activity, mini-open and endoscopic techniques have been developed. Endoscopic fasciotomy enables an earlier return to physical activity, with smaller scars and better aesthetic results (15). Regardless of the surgical technique, the rate of symptom resolution and return to activity varies from 50% to 90% (2, 3, 5). Thus, surgical treatment cannot be considered as a definitive management, especially for young adults (16).

In our case report, the patient showed a very transient regression of pain and functional discomfort following the surgical procedure, with symptoms reappearing as early as 10 weeks post-surgery. Despite this, post-exertion intra-muscular pressure measurements showed an overall decrease in pressure, with values at the lower limit of normal, therefore questioning the relationship between compartment pressure values and clinical symptoms. Indeed, despite the reduction in intramuscular pressure, the patient still has difficulty playing the drums. This prompts us to consider whether other factors might be responsible in the recurrence of symptoms, such as the surgical technique, postoperative adhesions, or the rehabilitation protocol. Our patient has only benefited from self-administered stretching exercises, but physiotherapy treatment under the supervision of a professional physiotherapist treatment would certainly have been more effective for optimal muscle strengthening protocol, including work on speed and fine motor skills. The surgical technique should also be debated. If the gold standard should be the fasciotomy of all 4 compartments, our patient underwent surgery only on positive and symptomatic compartments (anterior deep and superficial and posterior). This could therefore lead to a more significant recurrence of symptoms. Finally, it is also important to consider whether, for a professional drummer that need fine motor skills and rapid return to performance, surgery should be considered as the best first line option.

Non-surgical alternatives should therefore be proposed, such as injection of botulinum toxin. A PubMed search on “compartment syndrome AND botulinum toxin” lists only 32 publications, 8 of which focusing on CECS. One study reports injection into the upper and lower limbs (17), and another reports an injection into the hand (18). In 2 studies, patients underwent surgery prior to botulinum toxin injection (16, 18). The 36 patients identified in the other 6 publications benefited from botulinum toxin injection as first-line treatment. Overall, of the 38 patients treated by injection, 24 remained asymptomatic and continued their activities pain-free, 4 reported complete post-injection efficacy with recurrence within a median of 5 months, 5 reported partial initial efficacy with recurrence within a median of 2.25 months, and 5 remained painful. As in the study by (17) we noted a very good efficacy of botulinum toxin at 3 months post-injection, in terms of pain, stiffness and functional discomfort. There was also a clear reduction in post-exertion muscle pressures, with values within the normal range. Follow-up at 3 and 5 months post-injection also suggested a dose-dependent efficacy of botulinum toxin, with prolonged efficacy at 5 months with higher doses. In the available literature (17) results seem to be similar, with a recurrence of symptoms from 5 months post-injection. These results appear to be consistent with our case report where botulinum toxin injections seem to led to a reduction in post-exercise intramuscular pressure and increasing doses may result in more lasting effectiveness of the injections. Improvement in discomfort was subjectively assessed by the patient, but specific assessment scales could have provided a more objective and quantifiable description of the discomfort.

Even though there are no recommendations concerning the doses to be injected in this indication, it is generally assumed that patients with CECS should be injected with 1/3 of the dose used for patients suffering from neurological spasticity (19). Our study suggests that the administered doses should rather be adjusted according to the size of the muscle, the intramuscular pression and the patient's symptomatology. We decided to treat only the symptomatic muscles described by the patient. The only side effect reported by the patient in our study was a transient muscle weakness during the first month post-injection, which regressed completely within 1 month. Muscle weakness constitutes the main side effect due to the action of the botulinum toxin. This observation is similar to others studies using botulinum toxin injection in CECS and seems isolated (18, 20). Botulinum toxin should therefore be considered a treatment of choice combined with an adapted rehabilitation protocol. It could be proposed as a first-line treatment, depending on the patient's wishes (with a clear explanation of the risks and benefits of each technique), or, just like in our case, in the event of postoperative recurrence to avoid further surgery. Indeed, repeated surgeries favor the risk of postoperative complications such as hematomas, nerve damage, scar tissue adhesions, and infection. The toxin should also be considered to prevent excessive time off work for athletes or manual laborers. Of course, this single subject design inherently restricts any generalizability of the findings, and further studies are needed.

## Conclusion

Our case report suggests the efficacy of botulinum toxin injections, in case of surgical recurrence, on stiffness, pain and functional limitation in a patient with upper limb CECS. The average duration of efficacy was found to be up to 5 months post-injection, and varied according to the dose administered. The toxin also helps to reduce post-exertion intra-muscular pressure, although the relationship between symptom intensity and pressure value has yet to be demonstrated. The main side effect was a transient muscle weakness in the month following the toxin injection, which completely subsides within 1 month. Our case illustrates the potential of botulinum toxin in the treatment of CECS as a good therapeutic alternative to be explored in the event of recurrence or persistence of post-surgical pain and functional lilmitatiuon. Moreover, it could also be considered when patients desire conservative treatment, or as a transitional treatment before surgery for patients wishing to get through a period of competition or intense activity without having to stop their activities.

## Data Availability

The original contributions presented in the study are included in the article/Supplementary Material, further inquiries can be directed to the corresponding author.
